# Cultural and morphological divergence of Darwin’s cactus finches (*Geospiza scandens*) across Galápagos Islands

**DOI:** 10.1093/biolinnean/blaf098

**Published:** 2025-10-03

**Authors:** Melanie Kaluppa, Jefferson García-Loor, Alper Yelimlieş, Çağlar Akçay, Sonia Kleindorfer

**Affiliations:** 1 Konrad Lorenz Research Center for Behavior and Cognition, Core Facility of the University of Vienna, Grünau im Almtal 4645, Austria; 2 Department of Behavioral and Cognitive Biology, University of Vienna, Vienna 1030, Austria; 3 School of Life Sciences, Anglia Ruskin University, Cambridge, UK

**Keywords:** acoustic characteristics, allopatry, cultural evolution, Darwin’s finches, morphology, song, speciation

## Abstract

Understanding the divergence of cultural traits, such as bird song, provides crucial insights into evolutionary processes. In geographically isolated populations, the divergence of traits associated with mate choice can lead to further genetic separation. In this study, we investigate divergence in song syllable types, acoustic structure (e.g. kilohertz frequency), singing behaviour (e.g. number of syllables per song), and morphology in two allopatric populations of Darwin’s cactus finch (*Geospiza scandens*) on Floreana and Santa Cruz Islands in the Galápagos Archipelago. Using song recordings of 50 males, we identified 25 syllable types, with no overlap between islands, indicating a complete divergence in syllable repertoires. Syllables of Floreana and Santa Cruz males diverged in the acoustic space largely owing to broader frequency bandwidths on Floreana. Moreover, Floreana males had smaller beaks than Santa Cruz males. Despite acoustic and morphological divergence, singing behaviours, such as syllable repetition rate and number of syllables per song, did not differ significantly. Cultural processes, including drift and transmission biases, and selection on morphology could have contributed to the observed acoustic divergence. This study adds to a growing literature on the role of geographical separation in the accumulation of both cultural and morphological divergence between populations. Future research could interrogate speciation scenarios in cactus finches if populations cease to interbreed after secondary contact.

## Introduction

Allopatric speciation, the process by which geographically isolated populations diverge into distinct species, is a cornerstone of evolutionary biology (Mayr 1942). Physical barriers restrict gene flow between populations, which can lead to independent evolutionary pathways and the accumulation of morphological and behavioural differences. When such differences influence mate choice, they can contribute to reproductive isolation and, ultimately, speciation (Mayr 1963, Coyne and Orr 2004). Among behavioural traits, song in songbirds has been widely studied as a mechanism for maintaining barriers to gene flow. Birdsong plays a central role in both species recognition and mate choice (Catchpole and Slater 2008, Price 2008), and it reflects both cultural and genetic evolution, shaped by social learning and morphological constraints (Nowicki and Searcy 2005, Searcy *et al*. 2021, Vernes *et al*. 2021).

Bird song learning involves the cultural transmission of songs through a process of exposure, attention, imitation, and reproduction (Catchpole and Slater 2008). These processes offer numerous opportunities for error or modification of songs. The individual learning processes that lead to these changes can interact with social learning biases, which would lead to cultural evolution (Lipkind and Tchernichovski 2011, Aplin 2019, Williams and Lachlan 2022). It is worth noting that cultural evolution, defined as the transmission of behavioural traits across generations through social learning (Klump *et al*. 2021), is documented in vocal production learning systems beyond songbirds (oscine passerines), including at least two other orders of birds [parrots (Smith-Vidaurre *et al*. 2021) and hummingbirds (González and Ornelas 2014)], cetaceans (Garland and McGregor 2020), bats (Lin *et al*. 2015), and seals (Reichmuth and Casey 2014). Among these, bird song remains the most prominent model for cultural evolution in vocalizations (Whiten 2019).

Since the first systematic study of the songs of common chaffinch (*Fringilla coelebs*) by Marler (1952), geographical variation in birdsong has been documented across a wide range of species and spatial scales (Kroodsma 1985, Galeotti *et al*. 1996, Leader *et al*. 2000, Slabbekoorn et al. 2003, Reyes *et al*. 2024). Such variation typically results from a combination of geographical isolation and the dynamics of song learning (Podos and Warren 2007). Over time, cultural evolution can drive the emergence of new song types and the formation of local dialects, resulting in population-specific vocal patterns (Slater 2003, Parker *et al*. 2010, Aplin 2016). For example, isolated populations of swamp sparrows (*Melospiza georgiana*) maintain distinct song variants through cultural transmission, even in the absence of environmental or ecological differences (Lachlan *et al*. 2018). These vocal differences can act as prezygotic barriers by reducing interbreeding between populations, demonstrating how cultural evolution might contribute to reproductive isolation and, ultimately, speciation (Grant and Grant 1996, Colombelli-Négrel and Kleindorfer 2021).

In addition to the processes that underpin cultural evolution, in vocal-learning birds, morphological traits, such as those associated with beak shape, impose biomechanical constraints on sound production, creating a link between morphology and song structure (Podos and Nowicki 2004, Huber and Podos 2006, Christensen *et al*. 2006, Podos *et al*. 2009). Birds with larger beaks often produce slower or less complex songs, probably owing to biomechanical constraints that make it more difficult to filter out overtones in rapid or intricate vocalizations (Podos 2001). During singing, birds modify the length of their vocal tract (and consequently, its resonant frequencies) by opening and closing their beaks to emphasize different frequencies (Riede *et al*. 2006). Larger beaks, however, might be slower to open and close, limiting the speed and precision with which frequency modulation can occur. As a result, birds with larger beaks are often constrained to produce songs with narrower frequency bandwidths or slower repetition rates (Podos 2001, Podos *et al*. 2004, Huber and Podos 2006). Additionally, body size can also influence song characteristics (Ryan and Brenowitz 1985, Grant 1986, Brumm 2009, Martin *et al*. 2011). Body size affects syrinx volume, which, in turn, influences the minimum fundamental frequency of the song, as demonstrated in Darwin’s finches (Bowman 1983) and other bird species (Badyaev and Leaf 1997, Kirschel *et al*. 2009).

Darwin’s finches of the Galápagos Islands represent a particularly compelling model for studying the dynamics of speciation. Finch song typically consists of repeated renditions of one to two different syllable types, which are culturally transmitted. Juvenile males learn these syllables from a male tutor, usually their father or a nearby conspecific (Bowman 1979, 1983, Millington and Price 1985, Grant and Grant 1996, 2018, Christensen and Kleindorfer 2007, Peters and Kleindorfer 2018). Once learned, a finch’s syllable type(s) generally remain unchanged throughout its adult life, as observed in ground finches (Gibbs 1990, Grant and Grant 1996) and in male small tree finches (*Camarhynchus parvulus*) over a 2-year period (Christensen *et al*. 2006). Darwin’s finches exhibit song divergence across geographically separated populations (Grant and Grant 1997a, 1997b, Colombelli-Négrel and Kleindorfer 2021, Colombelli-Négrel *et al*. 2023), and this divergence has been linked to evolutionary and speciation processes (Grant and Grant 2009, Podos and Schroeder 2024). Beyond song divergence, Darwin’s finches show substantial morphological variation in beak and body size, not only between species but also between populations (Grant *et al*. 1985), and these correlate with vocal signal structures, such as frequency bandwidth and trill rate (Bowman 1979, Podos 2001). The combination of vocal variation and morphological diversity makes Darwin’s finches an excellent system for exploring the interplay between cultural drift, morphology, and acoustic divergence.

In the present study, we investigate potential differences in song by identifying the range of song syllable types and differences in acoustic structure of syllables (kilohertz frequency- and time-based features of individual syllables), male singing behaviour (number of syllables and syllable types per song, and syllable repetition rate), and male morphology across two geographically separated populations of Darwin’s cactus finches (*Geospiza scandens*) on Floreana and Santa Cruz Islands in the Galápagos Archipelago. First, we compile a library of syllable types within each population. Second, we compare the acoustic structure of syllables between the two island populations. Third, we analyse differences in male singing behaviour, including the number of syllables per song, the number of different syllable types used, and the rate of syllable repetition. Fourth, we assess morphological measurements across populations. If cultural and genetic isolation between these allopatric populations exists and has persisted long enough for divergence to occur, we predict: (i) both shared and unshared (population-specific) syllable types on each island; and (ii) inter-island differences in the acoustic structure of syllables. Additionally, if there are differences in song production or morphological constraints influencing song, we predict: (iii) inter-island differences in singing behaviour (specifically, in the number of syllable types per song, the number of syllables per song, and syllable repetition rate); and (iv) inter-island differences in morphological measurements.

## Materials and Methods

### Study sites and species

**Figure 1:**
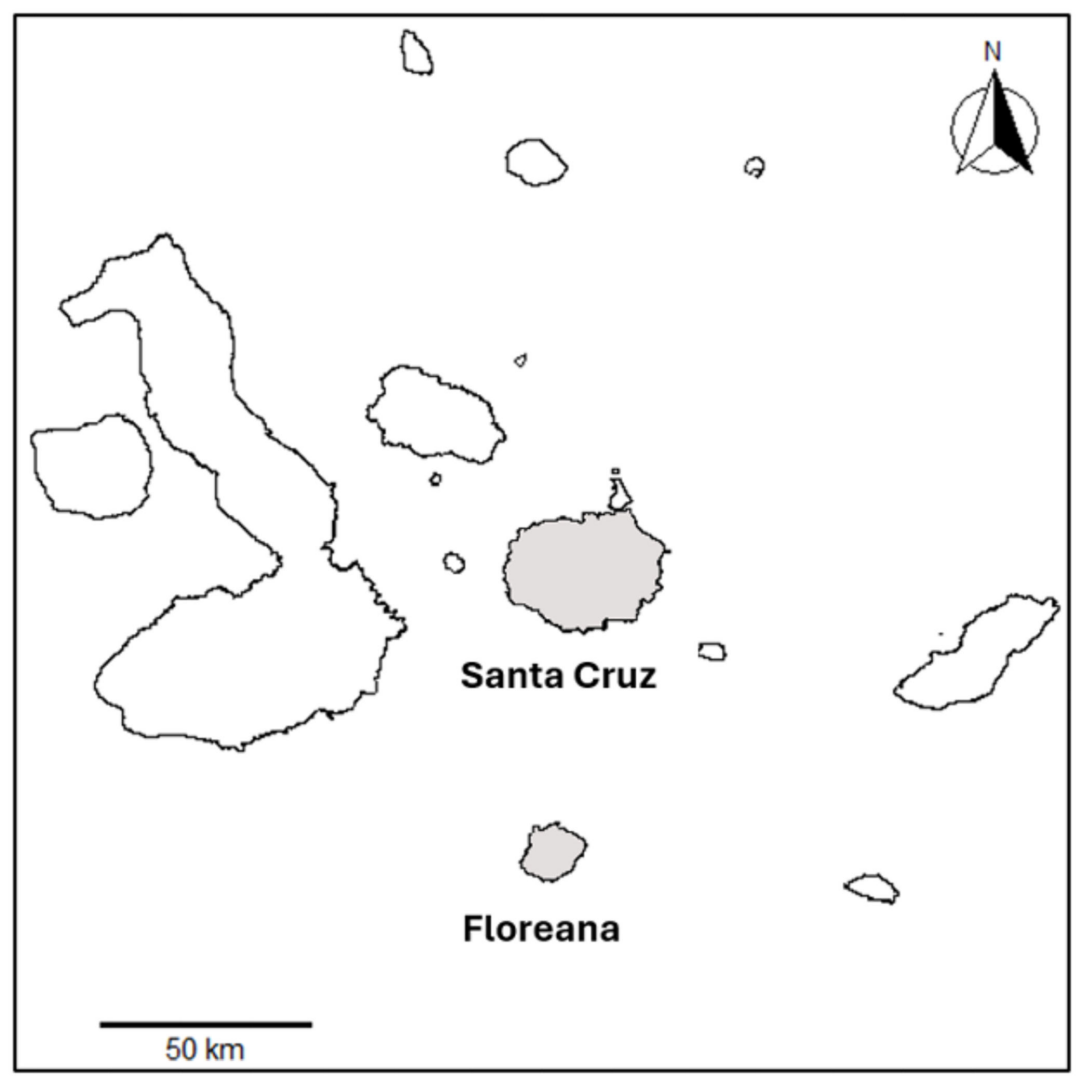
Map of the Galápagos Archipelago, with Santa Cruz and Floreana highlighted. The two islands are located ~47 km apart.

We collected acoustic data during the breeding season in March 2024, with recording sites located in the lowlands of Santa Cruz (Puerto Ayora, 0°44′S, 90°18′W; Garrapatero, 0°40′S, 90°13′W) and Floreana Island (1°,16′S, 90°29′W) in the Galápagos Archipelago (Fig. 1). The morphological data were collected between 2000 and 2024 as part of a long-term mist-netting programme (Supporting Information, Table S1). Located in the centre of the archipelago, Santa Cruz is the second largest island (986 km^2^) and lies ~47 km from the smaller Floreana Island (~173 km^2^). The arid lowland sites on both islands are primarily characterized by vegetation including shrubs, dwarf trees, and cacti from the genera *Opuntia* and *Jasminocereus* (Candelabra cactus) (Jackson 1993).

The common cactus finch (*Geospiza scandens*), one of 17 Darwin’s finch species (Passeriformes: Thraupidae), is a medium-sized ground finch (~22 g) found on most major Galápagos Islands, including Pinta, Santa Fé, Floreana, Santa Cruz, Isabela, Pinzón, Marchena, Santiago, and Rábida. Where absent, it is typically replaced by its close relatives; on Española, for example, by the Española cactus finch (Kleindorfer *et al*. 2019a, Jaramillo and Christie 2020). The common cactus finch inhabits dry scrublands and arid lowland woodlands, closely associated with prickly pear cacti (*Opuntia*) for food and nesting (Boag and Grant 1984). Its long beak, which is deep at the base and tapers to a point, is adapted for feeding on *Opuntia* pulp, seeds, and flowers (Abbott *et al*. 1977, Grant and Grant 1981, Millington and Grant 1983). Breeding typically begins in January or February, following the onset of heavier rains (Boag and Grant 1984). During this period, males build dome-shaped display nests and sing actively to attract mates and defend territories (Grant and Grant 1996).

### Recordings

To examine syllable type occurrence, acoustic structure, and singing behaviour between the two cactus finch populations, we recorded 25 males on each island, totalling 50 individuals. For 14 of the 25 males on Floreana and for all 25 males on Santa Cruz, we used playback of sympatric conspecific song to elicit singing (for song recording of the target male) when males were observed within their territory but were not vocalizing spontaneously. Playback consisted of a maximum of six songs per trial. The 1 min playback stimuli to elicit singing were broadcast with a JBL Clip 4 Bluetooth speaker (JBL Inc., USA) and consisted of six evenly spaced songs from the same individual, high-pass filtered at 1500 Hz, with normalized peak amplitude, and saved as 16 bit WAV files. The stimulus songs were recorded in the days prior, while the first individuals were establishing their territories. We accounted for playback use in our analyses to control for potential influences on measured song parameters. Recordings were made using a Zoom F3 Field Recorder (Zoom Corporation, Japan) and a Sennheiser MKE 600 directional microphone (Sennheiser electronic GmbH & Co. KG, Germany) at a sampling rate of 48 kHz and 32 bit float resolution. We aimed to record ≥10 high-quality songs per male (Floreana: mean = 11.7, SD = 2.94; Santa Cruz: mean = 14, SD = 3.20).

### Acoustic analysis

All audio recordings were visualized initially in Audacity v.3.4.2 (Audacity Team 2024) and exported at a 48 kHz sampling rate and 16 bit depth. Spectrograms were created using Raven Pro v.1.6.5 (K. Lisa Yang Center for Conservation Bioacoustics at the Cornell Lab of Ornithology 2024) with the following settings: Hann window, window length = 512, contrast = 50, and brightness = 50. For analysis, we selected songs with a high signal-to-noise ratio, yielding 642 total songs (Floreana, 292; Santa Cruz, 350). To define the start and end of each song, we classified a vocalization as a song if the time interval between the last syllable of the previous song and the first syllable of the next song was ≥1 s apart. Doubling this threshold from 1 to 2 s affected only 7 of 635 songs.

Each song was saved as an individual file and analysed in RSTUDIO (R Core Team 2024). We used the *warbleR* (Araya-Salas and Smith-Vidaurre 2017) and *ohun* (Araya-Salas and Smith-Vidaurre 2017) packages to apply energy-based detection through the *energy_detector()* function, which marked syllable start and end points within each song, based on energy and temporal features. To prevent low-frequency noise from affecting relative amplitude, a bandpass filter (1.5–8 kHz) was applied during detection. Detected syllables were verified manually on labelled spectrograms using *label_spectro()*, adjusting *threshold, smooth*, and *hold. time* as needed. This process identified a total of 2001 syllables (Floreana, 888; Santa Cruz, 1113).

To extract frequency- and time-based parameters for each syllable, we initially used *freq_ts()* to obtain the dominant frequency contour as a time series, from which minimum and maximum dominant frequencies, bandwidth, and slope were derived. We used a window length of 1024 points and 90% overlap. Given the sampling rate of 48 kHz, this resulted in a frequency resolution of ~47 Hz per bin and a temporal resolution of ~2.1 ms per analysis frame. Additional features were calculated via *spectro_analysis()*, including duration, mean/median frequency, SD, first/third quartile frequencies and times, interquartile ranges (frequency/time), skewness, kurtosis, and spectral, temporal, and spectrographic entropy (see package manual for details). Syllables were filtered (>1500 Hz), and peak frequency was derived using *meanspec()* from the *seewave* package (Sueur *et al*. 2008), representing the frequency of the highest amplitude of each sound file. Song duration was calculated from the start of the first to the end of the last detected syllable during energy-based syllable detection. Syllable repetition rate was computed by dividing the number of syllables per song by the song duration.

### Categorization of syllable types

Using the spectrograms generated in Raven Pro v.1.6.5, two researchers initially categorized the syllables into syllable types by consensus on the basis of structural similarities. To assess the generalizability of this categorization across researchers, five other raters were asked to classify the syllable types into categories. For this task, we used *spectro()* function of R from the *seewave* package to create spectrograms at the same scale, enhancing background-to-signal contrast. The raters received two printed spectrograms per syllable type, accompanied by instructions to create unique syllable type categories and/or match syllable type pairs for each island based on perceived image similarity. Inter-rater agreement was assessed by calculating the proportion of correctly matched syllable types between each rater and the original classification. The average proportional agreement across all raters was then calculated to provide an overall measure of inter-rater reliability.

### Morphological measurements

To examine morphological differences between the islands, we analysed measurements from 36 cactus finch males (17 from Floreana and 19 from Santa Cruz) at the time of capture and banding as part of our long-term mist-netting programme between 2000 and 2024 (Supporting Information, Table S1). The measured parameters included beak culmen length (tip of beak to base of feathers, in millimetres), beak width measured at the culmen (in millimetres), beak depth measured at the culmen (in millimetres), tarsus length (in millimetres), and flattened wing length (in millimetres). The birds in this sample were captured at the same sites as the sites from which the recordings stemmed.

### Data analysis

#### Acoustic structure

To investigate whether the acoustic structure of syllables differs between the two island populations, we conducted a principal component analysis using 21 frequency- and time-related measurements with *prcomp()* from the base *stats* package of R (R Core Team 2024). The first four principal components (PC1–PC4), each with an eigenvalue greater than one, collectively explained 86% of the variance and were selected for further analysis (Supporting Information, Table S2). PC factor loadings for all 21 acoustic variables were calculated (Supporting Information, Table S3).

PC1–PC4 were then used to conduct linear mixed models (LMMs), with each PC as the response variable, using *lmer()* from the package *lme4* (Bates *et al*. 2015). Island and playback use (yes/no) were included as fixed effects, whereas bird identity (ID) and syllable type were included as random effects.

#### Singing behaviour

To evaluate potential differences in male singing behaviour between the two islands, we analysed the number of syllable types per song, the number of syllables per song and the syllable repetition rate.

Generalized linear mixed models (GLMMs) from the *lme4* and *glmmTMB* packages (Bates *et al*. 2015, Brooks *et al*. 2017) were used for the number of syllable types per song and the number of syllables per song as response variables, with island and playback use included as fixed effects. Random effects differed by model: for the number of syllables per song, both bird ID and syllable type were included, whereas for the number of syllable types per song, only syllable type was used owing to negligible variance attributable to bird ID. A generalized Poisson error distribution was applied to the GLMM of the number of syllables to account for underdispersion. We modelled the number of syllable types as a binary response because our data contained only ones and twos. We subtracted one from each row and used a binomial response in our model. To assess variations in the syllable repetition rate between island populations, an LMM (package *lme4*) was applied, including island and playback as fixed effects and bird ID and syllable type as random effects.

#### Morphology

A principal component analysis was conducted on three beak measurements (length, width, and depth). The first principal component (PC1) had an eigenvalue greater than one and explained ~63% of the total variance; it was therefore used as the primary composite measure of beak size (Supporting Information, Table S4). A separate principal component analysis on body measurements (tarsus and wing length, in millimetres) identified PC1 as the main body size metric, explaining 62% of the variance (eigen-value = 1.23; Supporting Information, Table S4). Both PC1_beak and PC1_body were normally distributed and exhibited homogeneity of variance, as confirmed by the Shapiro–Wilk and Levene’s tests, respectively. We then performed independent two-tailed *t*-tests on PC1_beak and PC1_body to evaluate morphological differences in males between the two island populations.

## Results

### Syllable types

**Figure 2:**
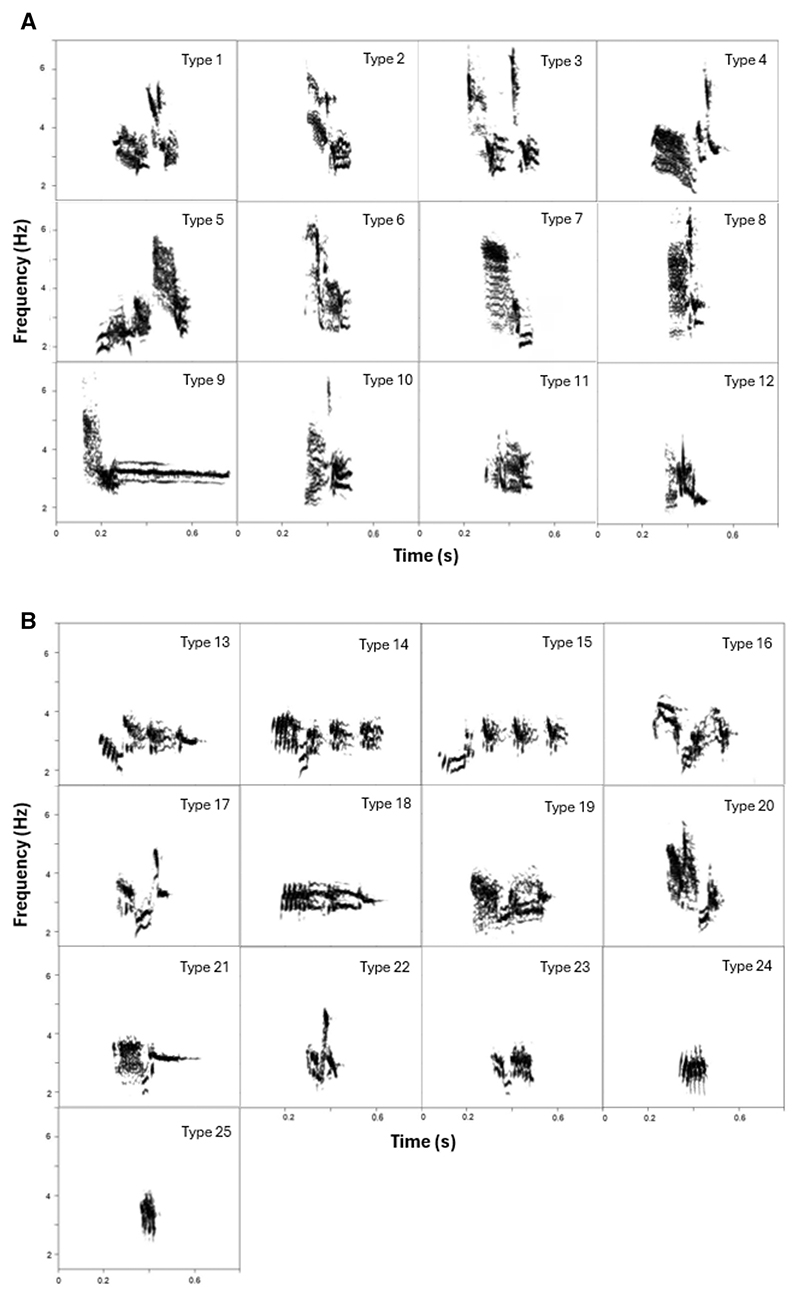
Examples of syllable types on Floreana (A) and Santa Cruz (B). Syllable types 1–12 were found exclusively on Floreana Island, whereas syllable types 13–25 were unique to Santa Cruz. No syllable types were shared between the islands.

Visual inspection of spectrograms identified 12 syllable types on Floreana and 13 on Santa Cruz, yielding a total of 25 syllable types. Classification was validated by five independent raters, resulting in an overall inter-rater agreement of 97%. Syllable types 1–12 were unique to Floreana Island, whereas types 13–25 were exclusive to Santa Cruz Island. No syllable types were shared between the two island populations (Fig. 2).

Of the 50 recorded males, 7 individuals (14%) produced songs containing two distinct syllable types, occurring in 12%–100% of their songs. The remaining 43 males (86%) produced songs consisting of only one syllable type. Supporting Information, Figure S1 presents spectrogram examples of songs from males with two syllable types. On Floreana Island, 5 of 25 males exhibited songs with two syllable types, in comparison to 2 of 25 males on Santa Cruz Island (Supporting Information, Table S5).

On Floreana Island, syllable type 2 occurred in the same song as syllable type 11 in two males, whereas three other males sang only syllable type 2 in their songs. On Santa Cruz, syllable type 13 occurred in the same song as syllable type 24 in one male and was also used by another male who sang only that syllable type. The syllable types 14 and 15 were produced separately in different songs by two males each, and both types occurred within the same song in one male.

On Floreana Island, half of the syllable types (6 of 12) were each produced by two to five males, whereas the remaining six syllable types were each sung by only one male (Table 1). On Santa Cruz Island, more than half of the syllable types (8 of 13) were each produced by two to six males, with the remaining five syllable types each sung by a single male (Table 1).

### Differences in the acoustic structure of the syllables across islands

The LMMs showed significant differences between islands for PC1 (higher factor loadings for frequency bandwidth) and PC4 (dominated by peak frequency). However, after applying a false discovery rate correction (Benjamini–Hochberg) to account for multiple comparisons across PC1–PC4, the inter-island difference in PC4 was no longer statistically significant (adjusted *P* = .113). A robust and significant difference remained for PC1. Specifically, males from Floreana produced syllable types with broader frequency bandwidths, attributable to more energy in higher frequencies, in comparison to males from Santa Cruz (PC1 difference estimate = 1.78, SE = .40, *t* = 4.42, *P* < .001). No significant differences were found between the islands for PC2 (time-related variables) or PC3 (spectral shape features). Playback had no significant effect on the acoustic features measured.

**Figure 3:**
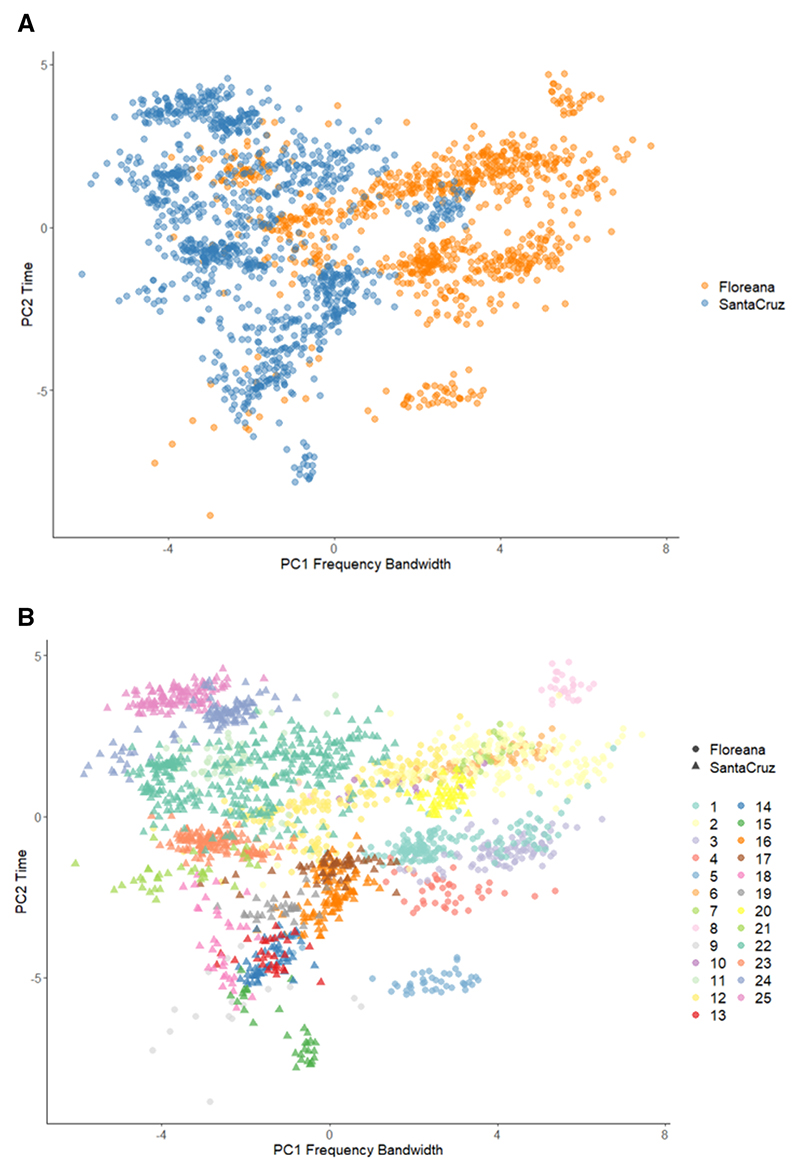
Variation in the acoustic space of syllable types based on the first two components, PC1 (dominated by frequency bandwidth) and PC2 (dominated by time-related variables), of the principal component analysis of acoustic structure. A, Santa Cruz males (blue circles) produced songs with a broader frequency bandwidth than Floreana males (orange circles). B, each syllable type [1–12 for Floreana (circles) and 13–25 for Santa Cruz (triangles)] clustered in an identifiable way according to frequency bandwidth (PC1) and time (PC2).

Visualization of PC1 and PC2 revealed not only differences between islands (Fig. 3A) but also variation among syllable types overall. Each syllable type clustered recognizably according to PC1 (frequency bandwidth) and PC2 (time) (Fig. 3B).

### Differences in singing behaviour

The models used to assess potential variations in male singing behaviour between the islands revealed no significant differences. Males did not differ in the number of syllable types per song (estimate = .43, SE = 3.58, *P* = .904), the number of syllables per song (estimate = −.004, SE = .09, *P* = .967), or the syllable repetition rate (estimate = −.08, SE = .25, *t* = −.306, *P* = .760) between the two islands.

### Differences in morphological measurements

Analysis of beak measurements (PC1_beak) showed significant differences between the islands. Cactus finch males from Santa Cruz had significantly larger beaks than males from Floreana (two-tailed *t*-test: *t* = −2.19, *N* = 17 of Floreana, *N* = 19 of Santa Cruz, *P* = .037; Table 2). There were no significant differences in body measurements (PC1_body) between the islands (two-tailed *t*-test: *t* = −1.32, *N* = 17 of Floreana, *N* = 19 of Santa Cruz, *P* = .197; Table 2).

## Discussion

To examine cultural and morphological divergence between two allopatric populations of Darwin’s cactus finches on Floreana and Santa Cruz Islands in the Galápagos Archipelago, we analysed the range of song syllable types, differences in the syllable acoustic structure, male singing behaviours, and male morphology. Our findings support the predictions of cultural divergence in song and morphological constraints on song production, demonstrating clear differences between the two island populations. In compiling a syllable library for each population, we identified 25 syllable types across 50 males (25 from each island), with no shared syllable types between islands. Floreana males produced 12 unique syllable types, whereas Santa Cruz males produced 13 unique types. The absence of shared syllable types emphasizes the cultural divergence in song and supports the prediction that cultural isolation has resulted in distinct syllable repertoires in the two populations.

When populations become geographically isolated or experience bottlenecks, both genetic and cultural divergence can occur, potentially leading to song divergence within a species. The complete absence of shared syllable types between Floreana and Santa Cruz might reflect processes such as cultural innovation (the introduction of new syllables through mutations; Marler 1970, Mundinger 1980), cultural drift (random changes in song structure over time; Potvin and Clegg 2015, Williams 2021, Williams and Lachlan 2022), and social learning biases (Aplin 2016), all of which can drive rapid changes in acoustic traits (Aplin 2019). Young Darwin’s finches acquire their songs through socially mediated learning from nearby males, typically their fathers or close neighbours (Bowman 1979, 1983, Millington and Price 1985, Grant and Grant 1996, 2018, Christensen and Kleindorfer 2007, Peters and Kleindorfer 2018). This learning process is not error free, similar to findings in zebra finches (Böhner 1983, 1990, Zann 1990, Mann and Slater 1994). Vocal production learning involves multiple stages: initial exposure, memorization, and vocal practice leading to song crystallization (Immelmann 1969, Marler 1970, Marler and Peters 1982a,b, Podos *et al*. 2009), each susceptible to error owing to perceptual inaccuracies, memory decay, or variation in motor output (Marler and Tamura 1964, Grant and Grant 1996, Podos and Warren 2007). These transmission errors can accumulate and propagate within populations, leading to subtle but significant song modifications. Over generations, such changes can result in the emergence of new syllable types or distinct population-specific song patterns (Slater 2003, Parker *et al*. 2010, Aplin 2016, Williams 2021).

The cultural nature of song transmission also enables rapid adaptation to changing environmental and social contexts. Female Darwin’s finches often prefer males whose songs differ from those of their fathers, a preference that might help to reduce inbreeding (Grant 1984, Grant and Grant 1989, 1995, 1996, Gibbs and Grant 1989). This mechanism can also promote syllable diversity within populations, because males with uncommon or distinct song characteristics might gain a reproductive advantage (Colombelli-Négrel *et al*. 2023). As a result, song can be a flexible trait that evolves independently of genetic changes, highlighting the significant role of cultural evolution in the speciation and diversification of Darwin’s finches.

Our analysis also revealed significant differences in acoustic parameters between the two populations. Males from Floreana produced syllables with broader frequency bandwidths in comparison to males from Santa Cruz. These differences might be influenced by morphological constraints on song production, because beak size has been shown to limit the frequency range and syllable repetition rate. Birds with larger beaks are less capable of rapidly opening and closing their beaks, which affects their song characteristics (Podos 2001, Podos *et al*. 2004, Huber and Podos 2006). Consistent with this, males from Santa Cruz (which produced narrower frequency bandwidths with the same syllable repetition rate) had larger beaks than their Floreana counterparts. This suggests that the narrower frequency bandwidths in Santa Cruz males might represent a compensatory adaptation to biomechanical limitations associated with their larger beaks while keeping the syllable repetition rate the same. Thus, song divergence might emerge as a byproduct of morphological differences in beak size (Podos 2001, Badyaev *et al*. 2008). Apart from differences in beak size, males from Floreana and Santa Cruz showed no significant difference in overall body size. Also, no differences were found between the islands in the number of syllable types per song. However, seven individuals (14%) in our sample produced songs with two syllable types, indicating some variation in song complexity within each population. Despite the observed differences in beak size and some acoustic parameters, no significant differences were detected in the number of syllables per song or in the syllable repetition rates between the two populations. This suggests that although the populations diverge in syllable repertoires and acoustic structure, such as frequency bandwidth, their overall singing behaviour remains largely consistent. These findings suggest that the mechanisms driving song divergence are more closely related to syllable type and morphological factors rather than changes in singing behaviour itself.

In addition to cultural evolution, hybridization with the closely related medium ground finch (*Geospiza fortis*) might also play a role in driving morphological and acoustic divergence between the cactus finch populations on Floreana and Santa Cruz. Previous studies have shown that interbreeding between cactus finches and medium ground finches is not uncommon (Grant and Grant 1997a, Grant *et al*. 2003, 2004, Thackeray 2024). Medium ground finches are characterized by shorter and broader beaks compared with cactus finches. Hybrid offspring could exhibit altered beak morphology and modified song characteristics, potentially introducing additional variability into the populations (Peters and Kleindorfer 2018, Kleindorfer *et al*. 2019b). This hybridization could contribute to both morphological and acoustic divergence, further complicating the mechanisms underlying song variation in the cactus finch populations.

Although hybridization between cactus finches and medium ground finches is likely to occur on both islands, its effects might be more pronounced in smaller populations, such as the one on Floreana (Grant *et al*. 2003). Small populations are particularly vulnerable to hybridization with closely related species owing to limited mate choice and smaller gene pools (Grant and Grant 1997a). Introgressive hybridization, as documented in Darwin’s finches, can have profound effects on morphology and behaviour, including changes in beak shape and song characteristics (Grant *et al*. 2004, Kleindorfer *et al*. 2014, Peters and Kleindorfer 2018, Dudaniec *et al*. 2025). For the cactus finches on Floreana, the introduction of medium ground finch genes could accelerate divergence, leading to distinct differences in both morphological traits and acoustic parameters when compared with larger or more stable populations, such as those on Santa Cruz.

During data collection, we also observed a potential shift in nest site preference on Floreana. Nest monitoring revealed that although *Opuntia* cacti (typically the primary nesting habitat of the cactus finch) were present, only 3 of 25 cactus finch nests were found in *Opuntia*. Instead, most nests (15) were located in *Candelabra* cacti, and 7 were built in trees (e.g. *Palo Santo, Acacia*) or vines. In contrast, medium ground finches were frequently observed defending nests in *Opuntia* on Floreana, indicating possible competition or a shift in nesting preferences. On Santa Cruz, cactus finches were primarily observed defending *Opuntia*; however, comprehensive nest monitoring was not conducted on that island. The extent of this possible shift in nest site preference, in addition to the genetic differences across populations, will be investigated in future research.

## Conclusion

Our findings reveal clear differences in syllable repertoires, frequency bandwidths, and beak morphology between the two cactus finch populations, supporting the idea that both cultural and morphological factors could have shaped song divergence in this system. The absence of shared syllable types underscores the role of cultural evolution in song differentiation, while beak size constraints are likely to contribute to differences in syllable structure. Additionally, potential hybridization with medium ground finches might introduce further variation in song and morphology, particularly in the smaller Floreana population. Planned follow-up studies, incorporating genetic analyses and playback experiments, will provide deeper insights into the mechanisms driving these differences. Such research could not only enhance our understanding of evolutionary processes and speciation but also inform conservation strategies to protect the unique ecosystems of the Galápagos Islands.

## Supplementary Material

Supplementary data is available at *Biological Journal of the Linnean Society* online.

Suplementary

## Figures and Tables

**Table 1:** Acoustic structure of syllable types on Floreana (A) and Santa Cruz (B) Islands.

Syllable type	Percentage occurrence	Duration (s)*^a^*	Minimum dominant frequency (kHz)*^a^*	Maximum dominant frequency (kHz)*^a^*	Bandwidth (kHz)*^a^*	Peak frequency (kHz)*^a^*
**(A) Floreana**						
**1**	16.7 (5)	0.28 (0.02)	2.61 (0.12)	5.49 (0.61)	2.88 (0.69)	3.13 (0.19)
**2**	16.7 (5)	0.22 (0.04)	2.66 (0.08)	6.02 (0.53)	3.36 (0.53)	3.52 (0.89)
**12**	16.7 (5)	0.21 (0.03)	2.25 (0.08)	4.3 (0.53)	2.05 (0.53)	2.68 (0.56)
**11**	13.3 (4)	0.14 (0.03)	2.72 (0.14)	3.79 (0.30)	1.07 (0.28)	3.1 (0.29)
**3**	10.0 (3)	0.31 (0.02)	2.7 (0.12)	6.18 (0.28)	3.48 (0.32)	3.3 (0.09)
**5**	6.7 (2)	0.42 (0.01)	1.99 (0.18)	5.32 (0.15)	3.33 (0.25)	2.54 (0.15)
**4**	3.3 (1)	0.33 (0.02)	2.08 (0.13)	5.69 (0.25)	3.61 (0.31)	3.38 (0.22)
**6**	3.3 (1)	0.21 (0.01)	2.73 (0.03)	6.09 (0.18)	3.36 (0.19)	3.51 (0.14)
**7**	3.3 (1)	0.21 (0.01)	2.15 (0.04)	5.33 (0.10)	3.18 (0.12)	4.69 (0.75)
**8**	3.3 (1)	0.17 (0.01)	2.98 (0.30)	6.04 (0.68)	3.06 (0.55)	3.53 (0.36)
**9**	3.3 (1)	0.6 (0.06)	2.78 (0.13)	5.05 (0.27)	2.27 (0.31)	3.25 (0.06)
**10**	3.3 (1)	0.2 (0.01)	2.57 (0.12)	5.3 (0.72)	2.73 (0.74)	2.84 (0.23)
	100.0 (30)					^a^Mean (SD)
**(B) Santa Cruz**						
**22**	22.2 (6)	0.16 (0.03)	2.59 (0.26)	4.09 (0.65)	1.5 (0.75)	3.2 (0.18)
**16**	11.1 (3)	0.33 (0.03)	2.26 (0.18)	4.28 (0.15)	2.02 (0.18)	3.67 (0.50)
**23**	11.1 (3)	0.21 (0.02)	2.31 (0.11)	3.33 (0.12)	1.02 (0.15)	3.09 (0.09)
**13**	7.4 (2)	0.45 (0.03)	2.38 (0.12)	3.61 (0.30)	1.23 (0.25)	3.04 (0.08)
**14**	7.4 (2)	0.48 (0.02)	2.36 (0.09)	3.68 (0.06)	1.33 (0.11)	3.18 (0.16)
**15**	7.4 (2)	0.51 (0.05)	2.1 (0.16)	3.57 (0.16)	1.47 (0.25)	3.18 (0.12)
**17**	7.4 (2)	0.27 (0.02)	2.28 (0.16)	4.72 (0.30)	2.44 (0.32)	3.33 (0.17)
**18**	7.4 (2)	0.45 (0.04)	2.47 (0.29)	3.34 (0.07)	0.87 (0.24)	3.04 (0.14)
**19**	3.7 (1)	0.35 (0.01)	2.47 (0.05)	3.76 (0.12)	1.29 (0.13)	3.21 (0.20)
**20**	3.7 (1)	0.24 (0.01)	2.29 (0.09)	4.93 (0.10)	2.64 (0.10)	3.56 (0.52)
**21**	3.7 (1)	0.28 (0.02)	2.4 (0.11)	3.59 (0.12)	1.19 (0.17)	3.13 (0.03)
**24**	3.7 (1)	0.11 (0.01)	2.94 (0.23)	3.68 (0.33)	0.74 (0.25)	3.51 (0.32)
**25**	3.7 (1)	0.08 (0.01)	3.09 (0.17)	3.65 (0.13)	0.56 (0.19)	3.3 (0.08)
	100.0 (27)					^a^Mean (SD)

Data are the mean values (SD) of the measured parameters, including duration, minimum dominant frequency, maximum dominant frequency, bandwidth, and peak frequency. Data include 25 males per island, whereby five males had songs with two syllable types on Floreana Island, and two males had songs with two syllable types on Santa Cruz. Syllable types are listed in order of their percentage occurrence, with the number of males producing each syllable type indicated in parentheses.

**Table 2:** Morphological data for 17 Floreana and 19 Santa Cruz males collected between 2000 and 2024.

Morphological variable	Floreana	Santa Cruz	*P*-value
	Mean ± SEM	Mean ± SEM	
**Beak length**	19.5 ± 0.6	21.1 ± 0.4	
**culmen**			
**Beak depth**	9.3 ± 0.1	9.6 ± 0.1	
**Beak width**	8.0 ± 0.2	8.5 ± 0.2	
**PC1_beak**	−0.5 ± 0.4	0.5 ± 0.3	**.037**
**Tarsus length**	22.3 ± 0.3	22.4 ± 0.3	
**Flattened wing**	69.7 ± 0.5	71.2 ± 0.6	
**length**			
**PC1_body**	−0.3 ± 0.3	0.2 ± 0.2	.197

The table shows mean values and the SEM of beak size (length, depth, and width, in millimetres) and body size (tarsus and wing length, in millimetres) per island. Principal component analysis of beak measurements (PC1_beak) revealed significantly larger beaks in Santa Cruz males (*P* = .037), whereas body size shows no significant differences between islands.

## Data Availability

The data underlying this article are available in PHAIDRA, at https://doi.org/10.25365/phaidra.708.
