# Supplementary material for: Cultural and morphological divergence of Darwin’s cactus finches (Geospiza scandens) across Galápagos Islands
Source: Biol J Linn Soc Lond. Author manuscript; Available in PMC 2026 May 19. (PMC7619092; doi:10.1093/biolinnean/blaf098)
Supplement: Suplementary [file EMS213484-supplement-Suplementary.zip › Supplementry_Data/Supporting Information.docx]

| **Year** | **Island** | **Male ID** | **Beak length culmen** | **Beak depth** | **Beak width** | **Tarsus length** | **Flattened wing length** | **PC1_beak** | **PC1_body** |
| --- | --- | --- | --- | --- | --- | --- | --- | --- | --- |
| 2005 | Floreana | SK1140 | 20.2 | 9.2 | 8.2 | 21.4 | 69.0 | -0.34 | -1.01 |
| 2005 | Floreana | SK1150 | 21.2 | 10.1 | 9.3 | 21.7 | 68.5 | 1.68 | -0.98 |
| 2005 | Floreana | SK1242 | 19.6 | 10.0 | 8.7 | 20.7 | 68.0 | 0.77 | -1.74 |
| 2005 | Floreana | SK1371 | 21.0 | 9.6 | 8.7 | 23.0 | 72.5 | 0.64 | 1.02 |
| 2016 | Floreana | SK3616 | 20.0 | 9.3 | 7.8 | 24.0 | 66.0 | -0.60 | -0.34 |
| 2016 | Floreana | SK3631 | 19.0 | 8.7 | 7.6 | 23.7 | 74.0 | -1.58 | 1.90 |
| 2016 | Floreana | SK3749 | 21.5 | 9.5 | 8.7 | 24.0 | 70.0 | 0.63 | 0.87 |
| 2016 | Floreana | SK3793 | 18.5 | 8.8 | 7.5 | 23.4 | 69.0 | -1.66 | 0.21 |
| 2020 | Floreana | SK3796 | 27.3 | 9.3 | 8.3 | 22.1 | 68.0 | 1.26 | -0.89 |
| 2020 | Floreana | SK3921 | 19.1 | 9.2 | 8.2 | 22.9 | 72.0 | -0.56 | 0.81 |
| 2020 | Floreana | SK3930 | 16.5 | 8.5 | 8.2 | 23.0 | 68.0 | -1.80 | -0.34 |
| 2020 | Floreana | SK3699 | 21.0 | 9.7 | 8.5 | 23.4 | 72.0 | 0.58 | 1.11 |
| 2024 | Floreana | SK5218 | 17.7 | 10.7 | 8.85 | 20.2 | 67.0 | 1.24 | -2.35 |
| 2024 | Floreana | SK5232 | 17.7 | 9.5 | 7.4 | 20.4 | 70.1 | -1.18 | -1.29 |
| 2024 | Floreana | SK5235 | 16.5 | 8.6 | 6.0 | 22.1 | 69.0 | -3.47 | -0.59 |
| 2024 | Floreana | SK5239 | 18.5 | 9.1 | 6.7 | 21.7 | 72.0 | -1.99 | 0.08 |
| 2024 | Floreana | SK5240 | 16.4 | 8.9 | 7.1 | 21.7 | 69.0 | -2.30 | -0.83 |
| 2000 | Santa Cruz | SC1 | 21.5 | 10.2 | 9.5 | 21.9 | 71.0 | 2.00 | -0.10 |
| 2000 | Santa Cruz | SC2 | 20.3 | 9.8 | 9.3 | 21.3 | 73.5 | 1.19 | 0.29 |
| 2001 | Santa Cruz | SC3 | 20.8 | 9.4 | 9.0 | 22.5 | 70.0 | 0.63 | -0.04 |
| 2001 | Santa Cruz | SK323 | 19.1 | 8.4 | 7.6 | 20.5 | 68.5 | -1.87 | -1.71 |
| 2002 | Santa Cruz | SC4 | 22.0 | 10.1 | 9.4 | 22.0 | 71.0 | 1.92 | -0.04 |
| 2002 | Santa Cruz | SC5 | 20.5 | 9.6 | 9.2 | 20.9 | 71.0 | 0.94 | -0.71 |
| 2004 | Santa Cruz | SK556 | 19.1 | 10.5 | 8.8 | 21.3 | 70.0 | 1.27 | -0.77 |
| 2004 | Santa Cruz | P556 | 21.8 | 9.4 | 9.2 | 23.8 | 73.5 | 0.99 | 1.81 |
| 2016 | Santa Cruz | SC6 | 25.6 | 9.1 | 8.4 | 23.5 | 70.0 | 0.79 | 0.57 |
| 2016 | Santa Cruz | SK3570 | 21.7 | 9.6 | 7.9 | 23.0 | 74.0 | 0.13 | 1.47 |
| 2016 | Santa Cruz | SK3571 | 22.2 | 10 | 9.0 | 23.3 | 72.0 | 1.53 | 1.05 |
| 2016 | Santa Cruz | SK3573 | 22.3 | 9.9 | 8.5 | 23.5 | 76.0 | 1.04 | 2.38 |
| 2016 | Santa Cruz | SK3574 | 19.9 | 9.0 | 8.0 | 24.0 | 67.0 | -0.77 | -0.03 |
| 2016 | Santa Cruz | SK3575 | 23.0 | 9.9 | 8.8 | 23.0 | 72.0 | 1.42 | 0.87 |
| 2016 | Santa Cruz | SH1220 | 22.4 | 8.3 | 7.1 | 22.6 | 69.0 | -1.72 | -0.28 |
| 2016 | Santa Cruz | SK3786 | 19.2 | 9.8 | 8.2 | 20.3 | 72.0 | 0.08 | -0.77 |
| 2016 | Santa Cruz | SK3787 | 19.8 | 9.7 | 7.2 | 21.4 | 69.0 | -0.71 | -1.01 |
| 2016 | Santa Cruz | SK3751 | 19.9 | 9.8 | 89.2 | 23.1 | 74.0 | 0.22 | 1.53 |
| 2016 | Santa Cruz | SK3763 | 20.0 | 9.5 | 7.8 | 22.8 | 69.0 | -0.39 | -0.16 |

**Table S1.** Morphological measurements of 36 male cactus finches from Floreana and Santa Cruz collected between 2000 and 2024. PC1_beak represents the first principal component from beak measurements (length, depth, width in mm), while PC1_body reflects the first principal component from body measurements (tarsus and flattened wing length in mm).

**Table S2.** Eigenvalues, proportion and cumulative variances for the first 4 components of the principal component analysis of the frequency- and time-related measurements.

| **PC** | **Eigenvalue** | | **Proportion variance** | | **Cumulative variance** | |
| --- | --- | --- | --- | --- | --- | --- |
| PC1 | 8.61 | 0.41 | | 0.41 | |  |
| PC2 | 6.08 | 0.29 | | 0.70 | |  |
| PC3 | 2.20 | 0.10 | | 0.80 | |  |
| PC4 | 1.18 | 0.06 | | 0.86 | |  |

**Table S3.** Factor loadings for the first 4 principal components from the PCA of 21 acoustic variables. Shading highlights the highest loadings for each PC (values > 0.3).

| **Factor** | **PC1_freq_bandwidth** | **PC2_time** | **PC3_spectral_shape** | **PC4_peak_freq** |
| --- | --- | --- | --- | --- |
| Duration (s) | -0.08 | 0.38 | 0.13 | 0.12 |
| Minimum dominant frequency (kHz) | 0.04 | -0.25 | 0.27 | 0.04 |
| Maximum dominant frequency (kHz) | -0.31 | -0.04 | 0.11 | -0.16 |
| Bandwidth (kHz) | -0.31 | 0.04 | 0.02 | -0.17 |
| Peak frequency (kHz) | -0.08 | -0.08 | 0.29 | 0.53 |
| Slope | 0.16 | 0.15 | 0.02 | -0.36 |
| Mean frequency (kHz) | -0.29 | -0.15 | 0.21 | -0.04 |
| Standard deviation (kHz) | -0.30 | -0.02 | -0.08 | -0.31 |
| Frequency median (kHz) | -0.25 | -0.17 | 0.23 | 0.18 |
| Frequency Q25 (kHz) | -0.13 | -0.21 | 0.43 | 0.17 |
| Frequency Q75 (kHz) | -0.31 | -0.10 | 0.11 | -0.16 |
| Frequency IQR (kHz) | -0.31 | -0.03 | -0.05 | -0.25 |
| Time median (s) | -0.08 | 0.37 | 0.13 | 0.04 |
| Time Q25 (s) | -0.10 | 0.35 | 0.09 | 0.05 |
| Time Q75 (s) | -0.09 | 0.38 | 0.13 | 0.08 |
| Time IQR (s) | -0.08 | 0.37 | 0.14 | 0.10 |
| Skew | 0.22 | 0.02 | 0.41 | -0.33 |
| Kurtosis | 0.16 | 0.02 | 0.45 | -0.39 |
| Spectral entropy | -0.31 | 0.05 | -0.17 | 0 |
| Time entropy | 0.16 | -0.32 | 0 | -0.02 |
| Spectrographic entropy | -0.29 | -0.12 | -0.2 | -0.02 |

**Table S4.** Loading coefficients of the principal component analysis of the beak and body measurements.

| **Factor** | **Loading coefficient to PC1_beak** |  | **Factor** | **Loading coefficient to PC1_body** |
| --- | --- | --- | --- | --- |
| Beak length culmen | 0.45 |  | Tarsus length | 0.71 |
| Beak depth | 0.59 |  | Flattened wing length | 0.71 |
| Beak width | 0.67 |  |  |  |
|  |  |  |  |  |
| Eigenvalue | 1.90 |  | Eigenvalue | 1.23 |
| %Variance | 63% |  | %Variance | 62% |

**Table S5.** Details of seven Darwin’s cactus finch males (of 50 males recorded during 2024) that produced a song with two syllable types; all other males (86 %) had song with one syllable type. The table includes male ID and Island, the number of song recordings (n) per male, the percentage of songs with one syllable type versus two syllable types, the syllable type combinations, and three syntax examples for each male.

| **Male ID** | **Island** | **#Recordings (n)** | | **% Song with 1 syllable type** | | **% Song with 2 syllable types** | | **Syllable types present** | **Syntax examples** |
| --- | --- | --- | --- | --- | --- | --- | --- | --- | --- |
| Male 1 | Floreana | 17 | 88 | | 12 | | 6 & 11 | | 6-6-6-6; 11-11-11; 6-6-11-11-6-6 |
| Male 2 | Floreana | 14 | 29 | | 71 | | 7 & 11 | | 7-7; 7-11-11-11-11; 7-7-11-11-11-11-7-7 |
| Male 3 | Floreana | 10 | 0 | | 100 | | 10 & 8 | | 10-8-8-8; 10-8-8-8-8; 10-8 |
| Male 4 | Floreana | 9 | 0 | | 100 | | 11 & 2 | | 11-2-2-2-2-2-2; 11-2-2-2-2-11-2; 11-2-2-2-2 |
| Male 5 | Floreana | 12 | 0 | | 100 | | 11 & 2 | | 11-2-2-2; 11-2-2; 11-2-2-2-2-2 |
| Male 6 | Santa Cruz | 23 | 78 | | 22 | | 24 & 13 | | 24-24-24; 13-13; 24-24-24-24-24-13 |
| Male 7 | Santa Cruz | 20 | 50 | | 50 | | 14 & 15 | | 14; 14-14-15; 14-15 |

1. **FLOREANA**


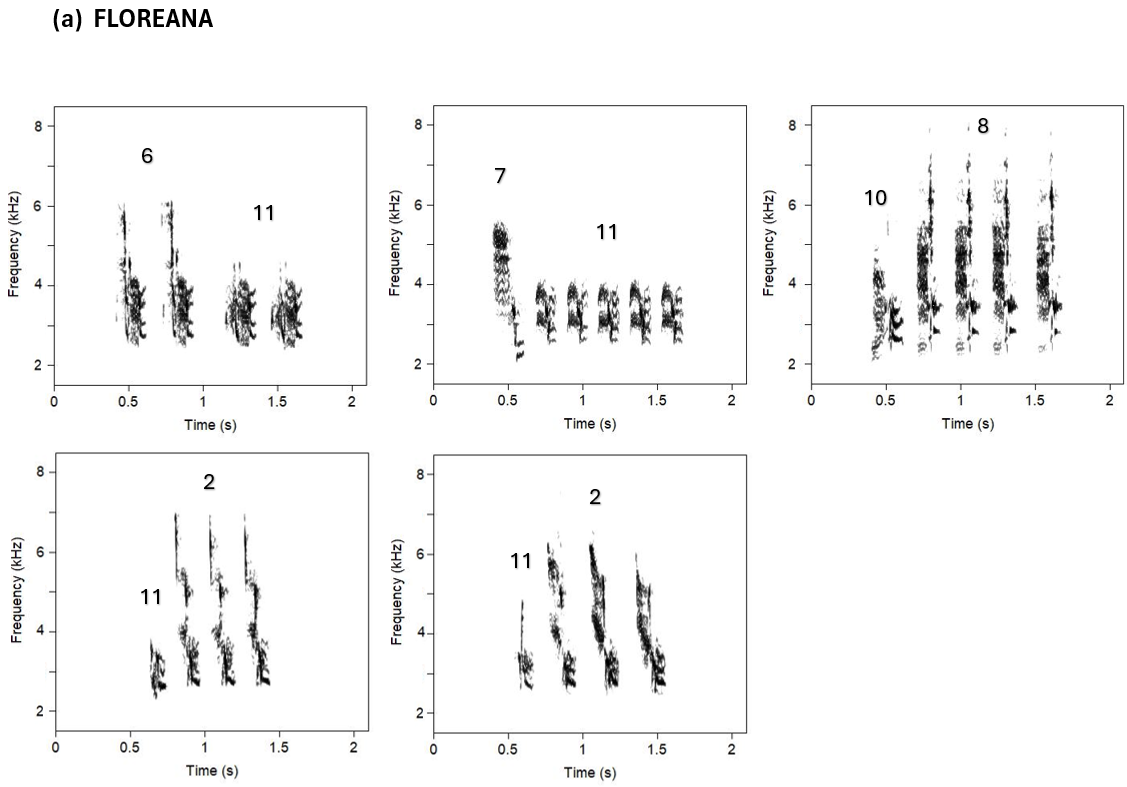


1. **SANTA CRUZ**


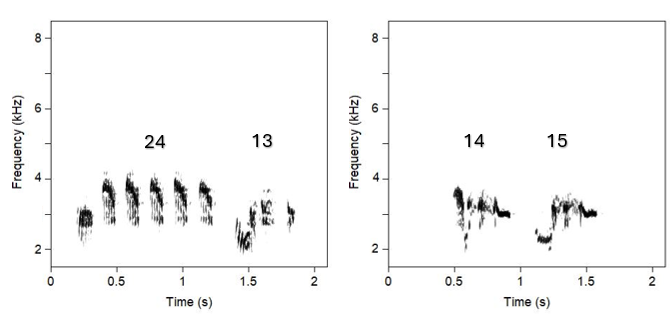


**Figure S1.** Spectrograms showcasing songs with two syllable types, including examples from the five males on **(A)** **Floreana** and the two males on **(B)** **Santa Cruz** that produced song with two syllable types in this study.
