# Supplementary figures and images for: Cultural and morphological divergence of Darwin’s cactus finches (Geospiza scandens) across Galápagos Islands
Source: Biol J Linn Soc Lond. Author manuscript; Available in PMC 2026 May 19. (PMC7619092; doi:10.1093/biolinnean/blaf098)

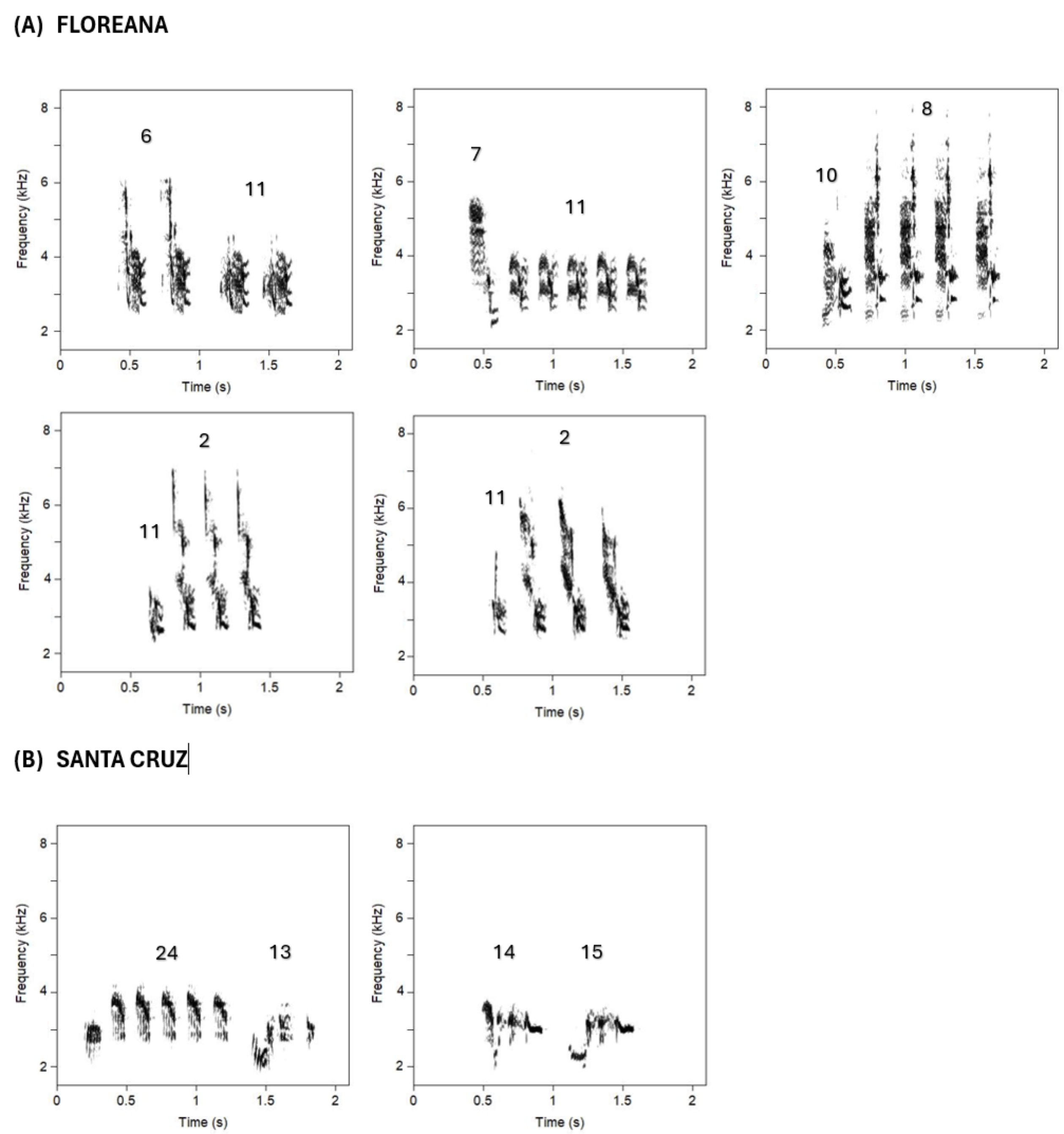

Supplement: Suplementary [file EMS213484-supplement-Suplementary.zip › Supplementry_Data/BJLS-7966 Figure S1.png]
